# Four-Year and Five-Developing-Stage Dynamic QTL Mapping for Tiller Number in the Hybrid Population of *Agropyron* Gaertn.

**DOI:** 10.3389/fpls.2022.835437

**Published:** 2022-02-24

**Authors:** Yonghe Che, Yutong He, Nan Song, Yanping Yang, Lai Wei, Xinming Yang, Yan Zhang, Jinpeng Zhang, Haiming Han, Xiuquan Li, Shenghui Zhou, Weihua Liu, Lihui Li

**Affiliations:** ^1^Hebei Key Laboratory of Crop Stress Biology, Qinhuangdao, China; ^2^College of Agronomy and Biotechnology, Hebei Normal University of Science and Technology, Qinhuangdao, China; ^3^Institute of Crop Sciences, Chinese Academy of Agricultural Sciences, Beijing, China

**Keywords:** *Agropyron* Gaertn., tiller number, unconditional QTL, conditional QTL, CP hybrid population

## Abstract

Tiller number (TN) is an important agronomic trait affecting gramineous crop yield. To understand the static and dynamic information of quantitative trait locus (QTLs) controlling TN of *Agropyron* Gaertn., both the unconditional and conditional quantitative trait loci (QTL) mapping of TN were conducted using a cross-pollinated (CP) hybrid population with a total of 113 plant lines from the cross between *Agropyron cristatum* (L.) Gaertn. Z1842 and *Allium mongolicum* Keng Z2098, based on the phenotypic data of TN at five developmental stages [i.e., recovering stage (RS), jointing stage (JS), heading stage (HS), flowering stage (FS), and maturity stage (MS)] in 4 years (i.e., 2017, 2018, 2020, and 2021) and the genetic map constructed of 1,023 single-nucleotide polymorphism (SNP) markers. Thirty-seven QTLs controlling TN were detected using two analysis methods in 4 years, which were distributed in six linkage groups. Each QTL explained 2.96–31.11% of the phenotypic variation, with a logarithum of odds (LOD) value of 2.51–13.95. Nine of these loci detected both unconditional and conditional QTLs. Twelve unconditional major QTLs and sixteen conditional major QTLs were detected. Three relatively major stable conditional QTLs, namely, *cQTN1-3*, *cQTN1-5*, and *cQTN4-1*, were expressed in 2020 and 2021. Meantime, two pairs of major QTLs *cQTN1-5* and *qTN1-4* and also *cQTN2-4* and *qTN2-3* were located at the same interval but in different years. Except for *qTN2-2* and *qTN3-5*/*cQTN3-5*, other thirty-four QTLs were first detected in this study. This study provides a better interpretation of genetic factors that selectively control tiller at different developmental stages and a reference for molecular marker-assisted selection in the related plant improvement.

## Introduction

*Agropyron* Gaertn. is a perennial forage grass, which is a wild relative of wheat with a P genome, and has the characteristics of high yield, good quality, strong stress resistance, and wide adaptability (Dewey, 1984; Asay and Johnson, 1990; Li and Dong, 1991). They are mainly distributed in arid and semiarid areas, such as Eurasia sandy temperate grassland, and in the northeast, northwest, Inner Mongolia, and other arid regions of China (Dewey and Asay, 1982; Che et al., 2014). Due to the advantages of withering late and returning early of *A.* Gaertn., and the withered grass that can also be eaten by animals, it has high feeding value and economic value, and it has been valued by the United States, Canada, and other animal husbandry developed countries. The root system of *A*. Gaertn. is well developed, so it shows strong drought tolerance. At the same time, *A*. Gaertn. also has a certain resistance to wheat susceptible diseases, such as stripe rust and powdery mildew, which is a high-quality genetic resource of wheat. Over the years, people have been committed to wide hybridization between perennial grasses and wheat, introducing excellent genes of wheat perennial grasses into wheat crops, and have made some progress (Liu et al., 2010). The hybridization between *A.* Gaertn. and wheat has been successfully achieved (Li and Dong, 1991), and several wheat cultivars having elite genes of P genome have been released in northern China. To sum up, *A*. Gaertn. is not only an excellent forage variety but also an important valuable donor of stress resistance and agronomic traits for wheat improvement (Alejandro et al., 2021).

The construction of a genetic linkage map is the basis of quantitative trait loci (QTL) mapping and molecular marker-assisted breeding for important traits of crops. The first genetic linkage map of tetraploid hybrid crested wheatgrass was constructed by a chromosome-doubling population, which used colchicine to introduce hybrid F_1_ seed (Jiang et al., 2015; Yu et al., 2015). Based on the map of tetraploid material, a total of 136 quantitative trait locus (QTLs) for 11 agronomy traits were detected (Yu et al., 2020). However, cross-pollinated (CP) plants, such as *A*. Gaertn., can only obtain heterozygous individuals caused by self-incompatible, and it is impossible to construct a population that can be inherited stably similar to the recombinant inbred line (RIL) or double haploid (DH) population of wheat (Ma et al., 2020; Jonathan et al., 2021). Thus, an alternative way was mentioned. Through the mapping method of “double pseudo-crossing,” some effective QTLs have been found in lots of forages (Jensen et al., 2005; Herrmann et al., 2006; Hirata et al., 2006). A genetic map of *Lolium perenne* was constructed, and QTL for resistance to stem rust was also detected using the “pseudo-crossing” F_1_ population (Pfender et al., 2011). Thus, we obtained the first high-density genetic linkage map of *A.* Gaertn. constructed using a CP population that contains 1,023 markers on seven linkage groups, with a total of 907.8 cm and an average distance of 1.5 cm between adjacent loci (Zhang et al., 2015). Based on this map, the major and stable QTL for plant height (PH; Che et al., 2020) and QTL for other characteristics of the spike (Che et al., 2018) in *A*. Gaertn. have been detected.

Tiller is the special branching method of gramineous plants and is closely related to yield. For example, the reduced-tillering wheat has yielded advantages when the water supply is less than 200 mm (Houshmandfar et al., 2019). The low expression of *TaPIN1* genes increases the tiller number (TN) as well as grain yield per plant of wheat (Yao et al., 2021). Thus, locating the gene that controlled TN will help improve the grain yield.

The TN is controlled by multiple genes (Haaning et al., 2020). At present, several candidate genes associated with TN have been reported in barley (Bai et al., 2021), and several single genes that control TN have been identified in wheat (Peng et al., 1998; Spielmeyer and Richards, 2004; Kuraparthy et al., 2007; Zhang et al., 2013; Wang et al., 2021). A validated, major QTL for effective tiller number (ETN) *Qetn-sau-1B.1* was located on chromosome 1BL of wheat, which could improve the ETN significantly, with the genetic map constructed of 55K array, simple sequence repeat (SSR), and kompetitive allele-specific PCR (KASP) markers (Liu et al., 2020). Another new, major, stably expressed QTL *Qetn-DW-4B.1* for ETN was identified on chromosome 4BL of tetraploid wheat (Chen et al., 2021). Moreover, the studies on TN were not only limited to the maturity stage, but also observed the growth stages of tillering dynamics that were dissected to find out the genetic information of dynamic expression of TN (Li et al., 2010). The dynamic QTL analysis of TN at four growth stages was conducted in wheat and predicted the candidate genes for TN (Ren et al., 2018). Although TN is important for yield and there are more studies on tillering dynamics, the understanding and investigation of the genetic basis of TN in *A.* Gaertn. are limited. Thus, the expression of QTL for TN at different developmental stages was investigated in this study combined with relevant phenotypic data and a genetic map. It could provide a foundation for TN genetic research of *A*. Gaertn. and related plant study.

## Materials and Methods

### Plant Material

A total of 113 individuals of the CP hybrid population, obtained by crossing between *Allium mongolicum* Z2098 (female, 2*n* = 14, PP) and *Agropyron cristatum* Z1842 (male, 2*n* = 14, PP), and two parents were transplanted to the farm of Hebei Normal University of Science and Technology in April 2014 (Che et al., 2018), and then clonal propagated from tillers and transplanted 115 ideal seedlings (including two parents) in April 2017. For controlling the planting density, the materials were transplanted in March 2020 again. The geographic location of the test site is 119°15′ E, 39°72′ N, with an average annual precipitation of 638.33 mm; frost-free period lasts up to 186 days; the soil is cinnamon soil, light loam, deep soil layer, and good permeability; it belongs to warm temperate, semi-humid continental climate. The designed planting row spacing in the experimental plot was 0.6 m, and the planting spacing was 0.4 m. Each material was designed with three replications and managed conventionally. The experiment was conducted in 2017, 2018, 2020, and 2021.

### Trait Phenotype and Data Analysis

The TN of the CP hybrid population and parents was investigated at each developmental stage in 2017, 2018, 2020, and 2021. The developmental stages were investigated every 2 days from the date of transplantation, and TN was investigated at each developmental stage in 2017; the survey was conducted from RS, JS, HS, and FS to MS including five developmental stages in 2018, 2020, and 2021 according to the standard ways (Li and Li, 2006). The SPSS 20.0 (SPSS, Chicago, IL, United States) was used for data statistics and analyzing genetic variation.

### Quantitative Trait Loci Mapping

Based on the CP hybrid population, the genetic map of the *Agropyron* whole genome was constructed using the specific-locus amplified fragment sequencing (SLAF-seq) to genotype single-nucleotide polymorphism (SNP) markers. The total length of the genetic map is 907.8 cm, including 1,023 SNP markers on seven linkage groups. JoinMap 4.0 (Stam, 1993) was used to construct the genetic linkage map (Zhang et al., 2015).

The QTL mapping was performed by GACD software (Li et al., 2008; Wang, 2009) with inclusive composite interval mapping. The walking speed for all QTL was set at 1.0 cM, *P* < 0.001, and logarithum of odds (LOD) > 2.5. When a chromosome interval met the above conditions, it was considered that there was a QTL affecting TN. When a QTL was detected with a contribution rate >10% in different environments, it was regarded as a major QTL, and the QTL that was detected in at least three different environments was defined as a stable QTL (Fan et al., 2015; Che et al., 2020). The name of the unconditional QTL is “q + TN + chromosome number + serial number” (Mccouch et al., 1997). The conditional QTL was named with “cQ + TN + chromosome number + serial number” to distinguish and describe. The QTL found in the same site in the chromosome was regarded as the same QTL in this study.

The conditional QTL analysis was according to the method described by Zhu (1995). The genetic effect of conditional QTL refers to the net genetic effect from one time to the other. For example, JS-RS was the net growth of TN phenotype value at RS-JS, and HS-JS was the net growth value at JS-HS. The genetic effect of unconditional QTL represents the total amount of genetic effect from sowing to the specified time.

## Results

### Phenotype Analysis in *Agropyron* Gaertn.

**FIGURE 1:**
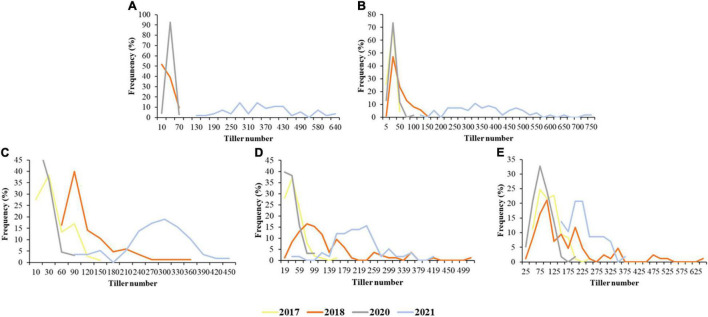
Frequency of tiller number (TN) at different stages in the *Agropyron* cross-pollinated (CP) population in 4 years. **(A–E)** Were the frequency of TN at the recovering stage (RS), jointing stage (JS), heading stage (HS), flowering stage (FS), and maturity stage (MS), respectively; the different colors for the different years.

The TN of the *Agropyron* CP hybrid population had been surveyed at RS-MS in 4 years. There were some differences in TN growth trends in 4 years, and TN also showed significant differences at each developmental stage of the year. TN increased slowly at the early growth stages and faster at the later growth stages in 2017 and 2018. TN experienced a gradual decrease and then a slow increase in 2020. Then, in 2021, TN increased at RS-JS started decreasing at JS-FS, and increased again at FS-MS. The stage of the most TN was MS in 2017, 2018, and 2020 but, in 2021, was JS due to special climate situations (warm winter in 2020 and summer waterlogging in 2021). In addition, there were great differences in TN among different individuals. The male parent had more TN than the female parent at the initial stage (JS), but the TN of the female parent was higher than that of the male parent at the later stage (FS and MS). The variation coefficient of TN in the CP hybrid population was 53.52–78.48, 77.48–98.42, 45.00–97.23, and 31.28–46.12% in 2017, 2018, 2020, and 2021, respectively (Table 1). TN in all 4 years showed a normal distribution, which was suitable for the QTL analysis (Figure 1).

**TABLE 1:** Tiller number (TN) of F_1_ population at different stages in *A.* Gaertn. under 4 years.

Year	Trait	Mean ± SD	Range	Coefficient of variation (%)	Skewness	Kurtosis	*W*-test
2017	TN at JS	11.63 ± 8.31	45	71.48	1.09	1.83	0.92
	TN at HS	21.86 ± 17.15	98	78.48	1.72	3.71	0.85
	TN at FS	36.46 ± 23.88	147	65.60	1.53	4.21	0.90
	TN at MS	71.29 ± 38.15	190	53.52	0.78	0.32	0.94
2018	TN at RS	15.13 ± 14.89	56	98.42	1.24	0.55	0.82
	TN at JS	39.82 ± 31.83	124	79.94	1.17	0.41	0.84
	TN at HS	76.59 ± 63.14	313	82.45	1.74	3.04	0.81
	TN at FS	121.82 ± 94.39	481	77.48	1.75	3.06	0.81
	TN at MS	148.66 ± 121.97	645	82.05	1.83	3.52	0.81
2020	TN at RS	23.11 ± 8.96	73	45.00	1.45	4.74	0.92
	TN at JS	13.95 ± 10.75	78	88.25	2.18	7.79	0.83
	TN at HS	11.87 ± 8.56	50	96.97	1.39	2.14	0.86
	TN at FS	27.87 ± 20.35	83	97.23	1.11	0.78	0.88
	TN at MS	64.58 ± 38.13	207	88.89	0.68	−0.23	0.92
2021	TN at RS	342.05 ± 135.90	632	42.12	0.18	−0.10	0.98
	TN at JS	352.38 ± 148.59	801	46.12	0.75	0.41	0.97
	TN at HS	237.51 ± 77.50	417	35.11	0.90	−0.68	0.96
	TN at FS	209.69 ± 68.50	432	36.43	1.57	0.26	0.96
	TN at MS	218.16 ± 54.07	311	31.28	0.41	0.52	0.98

### Unconditional Dynamic Quantitative Trait Loci Analysis of Tiller Number

**FIGURE 2:**
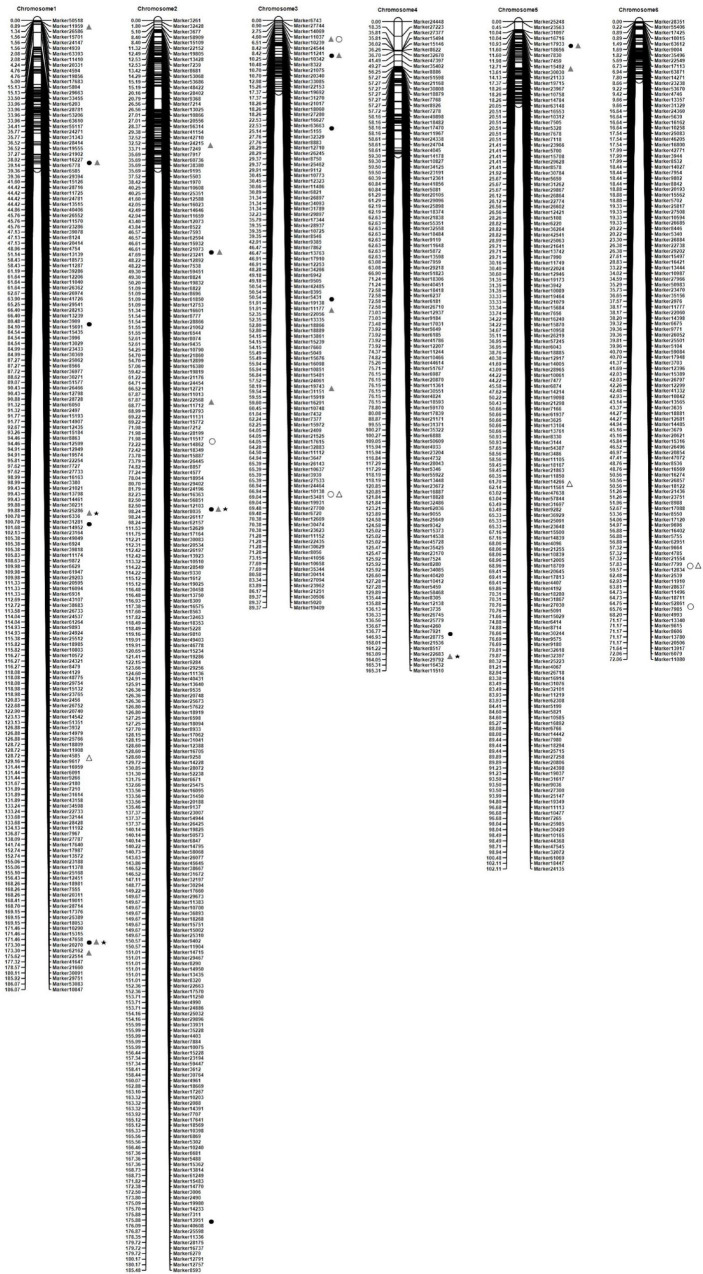
Dynamic identifications of quantitative trait loci (QTLs) controlling TN in different periods in the *Agropyron* CP hybrid population. ⬤, major unconditional QTL; ◯, no-major unconditional QTL; ▲, major conditional QTL; △, no-major conditional QTL; ★, QTL detected in 2 years.

**FIGURE 3:**
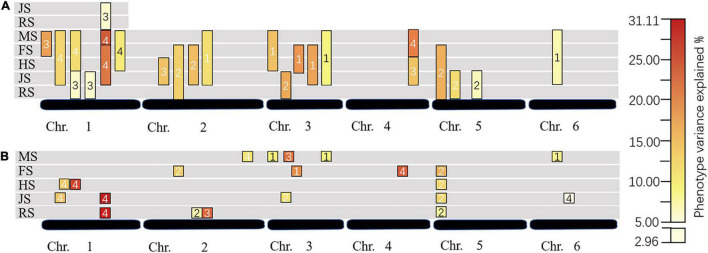
Dynamic QTL changes in different stages and years. **(A)** For conditional QTL; **(B)** for unconditional QTL; RS, JS, HS, FS, and MS for recovering stage, jointing stage, heading stage, flowering stage, and maturity stage, respectively; the numbers in the box: 1 for 2017, 2 for 2018, 3 for 2020, and 4 for 2021.

A total of seventeen QTLs controlling TN were discovered using unconditional QTL analysis in 4 years, which were located on six linkage groups except chromosome 7. There were four, four, five, one, one, and two QTLs from chromosome 1 to chromosome 6, respectively. The phenotypic variation explained (PVE) of a single QTL ranged from 2.96 to 31.11%, and the LOD value ranged from 2.51 to 13.95 (Table 2 and Figures 2, 3). Four, four, two, and seven QTLs were detected in 2017, 2018, 2020, and 2021, respectively. There were 70.59% (12/17) of the unconditional QTLs that were detected as major QTL (with PVE more than 10%).

**TABLE 2:** Unconditional quantitative trait locus (QTL) positioning of TN at different stages in *A.* Gaertn.

QTL	Stage	Position	Marker interval	LOD	Phenotypic variation explained (%)	Additive effect (female)	Additive effect (male)
*qTN1-1*	TJ4	39	Marker16227-Marker6778	7.22	12.57	104.15	–2.69
*qTN1-2*	TH4	81	Marker3909-Marker15691	4.65	12.19	40.55	–10.63
*qTN1-3*	TH4	101	Marker31281-Marker14952	8.88	26.32	–60.08	–7.69
*qTN1-4*	TR4	173	Marker47658-Marker20270	2.82	29.76	–46.86	3.79
	TJ4	173	Marker47658-Marker20270	13.93	31.11	–162.91	–4.43
*qTN2-1*	TF2	47	Marker21073-Marker23241	3.31	12.68	–36.59	–16.40
*qTN2-2*	TR2	72	Marker11517-Marker14862	2.51	7.02	–4.68	–2.79
*qTN2-3*	TR3	98	Marker12103-Marker8035	2.85	22.53	–1.43	2.62
*qTN2-4*	TM4	176	Marker13951-Marker40608	2.52	11.40	–22.92	–8.57
*qTN3-1*	TM1	5	Marker11037-Marker10239	3.07	9.39	12.16	1.37
*qTN3-2*	TJ2	9	Marker11241-Marker10342	2.89	10.58	9.97	3.35
*qTN3-3*	TM3	25	Marker63663-Marker5155	2.99	21.77	–2.36	2.49
*qTN3-4*	TF1	51	Marker5431-Marker19138	4.05	18.87	8.91	0.94
*qTN3-5*	TM1	67	Marker10138-Marker53481	2.66	8.25	12.79	–2.05
*qTN4-1*	TF4	142	Marker7921-Marker28775	3.23	23.14	24.26	–15.27
*qTN5-1*	TR2	11	Marker17933-Marker18656	2.78	7.73	2.39	1.16
	TJ2	11	Marker17933-Marker18656	2.92	12.31	4.22	–0.45
	TH2	11	Marker17933-Marker18656	2.78	11.79	8.01	2.98
	TF2	11	Marker17933-Marker18656	3.87	15.22	5.10	4.81
*qTN6-1*	T M1	58	Marker7799-Marker12834	2.56	7.90	–4.67	2.40
*qTN6-2*	TJ4	65	Marker52861-Marker7985	2.70	2.96	–19.93	6.70

*QTL named “q + trait + chromosome + number,” such as qTN1-1 indicating that the first QTL controlling TN, was located on chromosome 1; The numbers after stages means the stage of which year, 1 for 2017, 2 for 2018, 3 for 2020, and 4 for 2021.*

Four TN QTLs (*qTN1-4, qTN2-2, qTN2-3*, and *qTN5-1*) expressed at RS with the LOD value of 2.51–2.85, of which *qTN1-4* and *qTN2-3* were the major QTLs with 29.76 and 22.53% PVE, respectively. Five QTLs (*qTN1-1, qTN1-4, qTN3-2, qTN5-1*, and *qTN6-2*) expressed at JS were mapped, of which *qTN1-4* and *qTN5-1* were detected for the second time with the LOD values of 13.93 and 2.92 and the PVE of 31.11 and 12.31%, respectively. Also, *qTN1-1* and *qTN3-2* explained 12.57 and 10.58% phenotypic variation. Three major QTLs (*qTN1-2, qTN1-3*, and *qTN5-1*) at HS were located with the LOD value from 2.78 to 8.88, of which the *qTN5-1* was detected for the third time with 11.79% PVE. Four QTLs (*qTN2-1, qTN3-4, qTN4-1*, and *qTN5-1*) at FS were detected with the LOD value of 3.23–4.05. All of them were the major QTLs with 12.68–23.14% PVE, of which *qTN5-1* was detected for the fourth time at this stage. Five QTLs (*qTN2-4, qTN3-1, qTN3-3, qTN3-5*, and *qTN6-1*) at MS were detected with the LOD value of 2.52–3.07, of which *qTN2-4* and *qTN3-3* were the major QTLs with 11.40 and 21.77% PVE, respectively. Among the seventeen QTLs, *qTN5-1* was detected at four developmental stages (and as a major QTL at JS-FS), major QTL *qTN1-4* at two stages (RS-JS), and others once.

### Conditional Dynamic Quantitative Trait Loci Analysis for Tiller Number

In this study, we analyzed the expression of TN QTL at any two stages. No QTL was detected at RS-HS and RS-MS. A total of twenty conditional QTLs were detected by the conditional QTL analysis method, and a number of these QTLs distributed on chromosomes 1, 2, 3, 4, 5, and 6, were six, four, five, one, three, and one, respectively. Six QTLs were detected in each of the 4 years, respectively. There were 16 major QTLs and the single major QTL with PVE from 10.02 to 25.91%. Notably, three conditional major QTLs detected in 2020 (*cQTN1-3, cQTN1-5*, and *cQTN4-1*) were detected again in 2021 (Table 3 and Figures 2, 3).

**TABLE 3:** Conditional QTL positioning of TN at different stages in *A.* Gaertn.

QTL	Stage	Position	Marker interval	LOD	Phenotypic variation explained (%)	Additive effect (female)	Additive effect (male)
*cQTN1-1*	TMTF3	1	Marker11959-Marker26586	2.72	16.80	–1.59	–7.85
*cQTN1-2*	TMTJ4	39	Marker16227-Marker6778	8.18	11.46	–110.47	–3.76
*cQTN1-3*	TJTR3	100	Marker25286-Marker6336	2.76	6.02	–2.50	0.78
	TMTH4	100	Marker25286-Marker6336	2.85	10.59	27.71	–4.84
*cQTN1-4*	TJTR3	129	Marker4585-Marker9617	2.73	5.96	–2.51	0.56
*cQTN1-5*	TJTR3	173	Marker47658-Marker20270	2.95	6.43	–2.37	0.65
	TFTJ4	173	Marker47658-Marker20270	2.99	24.91	49.59	–11.12
	TMTJ4	173	Marker47658-Marker20270	13.95	24.10	159.58	–2.09
*cQTN1-6*	TMTH4	175	Marker62162-Marker22514	2.67	10.02	27.55	–1.46
*cQTN2-1*	THTJ3	33	Marker24215-Marker7249	2.57	12.95	–0.10	–1.45
*cQTN2-2*	TFTR2	47	Marker21073-Marker23241	3.41	12.11	–32.15	–12.98
*cQTN2-3*	TFTJ2	68	Marker22568-Marker11712	2.57	13.33	–24.94	–9.65
*cQTN2-4*	TMTJ1	92	Marker12103-Marker8035	2.54	11.10	7.19	–6.42
*cQTN3-1*	TMTH1	5	Marker11037-Marker10239	2.77	13.85	9.18	2.68
*cQTN3-2*	TJTR2	10	Marker11241-Marker10342	4.23	15.05	7.17	2.38
*cQTN3-3*	TFTH1	52	Marker11177-Marker22056	5.04	16.73	6.41	2.46
*cQTN3-4*	TFTJ1	59	Marker19743-Marker31151	3.54	15.64	7.43	0.81
*cQTN3-5*	TMTJ1	67	Marker10138-Marker53481	2.65	8.89	11.64	–1.51
*cQTN4-1*	THTJ3	164	Marker22683-Marker29792	2.88	12.98	0.93	–2.04
	TMTF4	164	Marker22683-Marker29792	2.63	19.18	–19.01	20.17
*cQTN5-1*	TFTR2	11	Marker17933-Marker18656	3.84	13.94	3.25	3.83
*cQTN5-2*	TJTR2	13	Marker15492-Marker30030	2.99	10.74	0.52	–1.12
*cQTN5-3*	TJTR2	62	Marker14266-Marker11561	2.51	7.75	–4.91	–1.21
*cQTN6-1*	TMTJ1	58	Marker7799-Marker12834	2.51	7.41	–3.37	1.98

*QTL named “cQ + trait + chromosome + number,” such as cQTN3-1 indicating that the first QTL controlling tiller in a period, was located on chromosome 3; The numbers after stages means the stage of which year, 1 for 2017, 2 for 2018, 3 for 2020, and 4 for 2021.*

With a start of RS, a total of eight conditional QTLs were detected. The highest number of QTLs were detected at RS-JS, with six in total, distributed on chromosomes 1, 3, and 5. The LOD value ranged from 2.51 to 4.23, and the PVE ranged from 5.96 to 15.05%. Two (*cQTN3-2* and *cQTN5-2*) of them were the major QTLs. Two major conditional QTLs (*cQTN2-2* and *cQTN5-1*) controlled TN at RS-FS were detected with the LOD values of 3.41 and 3.84 and the PVE of 12.11 and 13.94%. Starting with JS, a total of nine conditional QTLs were detected. Two major conditional QTLs (*cQTN2-1* and *cQTN4-1*) were detected at JS-HS. Three major conditional QTLs (*cQTN1-5, cQTN2-3*, and *cQTN3-4*) were detected at JS-FS, of which *cQTN1-5* was detected for the second time, with a larger PVE value of 24.91%. Five QTLs (*cQTN1-2, cQTN1-5, cQTN2-4, cQTN3-5*, and *cQTN6-1*) were located at JS-MS, of which *cQTN1-2, cQTN1-5*, and *cQTN2-4* were major, and *cQTN1-5* was detected for the third time, with the PVE of 24.10%. One (*cQTN3-3*) and three major QTLs (*cQTN1-3, cQTN1-6, and cQTN3-1*) were detected at HS-FS, and HS-MS, respectively. The *cQTN1-3* was previously expressed at RS-JS with a lower PVE (6.02%) than HS-MS (10.59%). Two major QTLs (*cQTN1-1 and cQTN4-1*) were found at FS-MS, while the *cQTN4-1* was also detected at JS-HS.

### Overlapping Unconditional and Conditional Quantitative Trait Locus

A total of nine intervals detected both conditional and unconditional QTLs. Seven pairs of conditional and unconditional QTLs with the same flanking markers were detected in the same year (Figures 2, 3). Among them, the unconditional major QTL *qTN5-1* was located at 11 cM on chromosome 5 (Marker17933–Marker18656), expressed at RS-FS, with a gradually increased PVE (total of 47.06%). Meanwhile, *cQTN5-1* was located at the same interval, and controlled TN at RS-FS, with 13.37% PVE. Also, two intervals were detected major conditional and unconditional QTLs that were expressed in different years. QTLs *cQTN2-4* and *qTN2-3* were located at Marker12103–Marker8035 on chromosome 2, of which *cQTN2-4* regulated TN at JS-MS in 2017, and *qTN2-3* was detected at RS in 2020. Another pair of QTL was located at Marker47658–Marker20270 on chromosome 1. The unconditional major QTL *qTN1-4* was detected at the two developmental stages of RS and JS in 2021, and conditional major QTL *cQTN1-5* was at RS-JS in 2020. The conditional QTL *cQTN1-5* was also expressed in 2021, which started from JS to FS and MS, respectively.

## Discussion

### Continuous Quantitative Trait Loci Associated With Tiller

A stable QTL for TN through the different environments is vital for marker-assisted selection in breeding varieties adapted to various ecological environments (Bilgrami et al., 2020). This study did not detect a stable QTL in more than three environments. This might cause by the difference of environments or climate in four different years (Campbell et al., 2003); or as a kind of perennial plant, the regrowth capacity of *A.* Gaertn. planted from 2014 to 2021 might be weakened year by year, and tiller ability has also declined. With the meta-analysis method, three stable marker-trait associations for maximum tiller in spring were detected on chromosomes 1B, 2B, and 6B of wheat in two different environments (Chen et al., 2017). Two stable QTLs for ratoon stunting disease resistance were detected in 2 years (You et al., 2021). The stable QTLs in the above mentioned studies were detected in 2 years or two environments. Similarly, the three major conditional QTLs, namely, *cQTN1-3, cQTN1-5*, and *cQTN4-1*, were detected in 2 years in this study, although the period they expressed was different in 2 years. Thus, these QTLs could be regarded as relatively stable QTL. Furthermore, conditional and unconditional QTLs detected in the same interval but in different years could also be considered as stable QTLs, such as *cQTN2-4* and *qTN2-3*. To sum up, a total of four stable QTLs were detected in this study.

In addition, a QTL had multiple effects that could improve the efficiency of assistant breeding (Wang et al., 2018). For example, the QTL controlling TN of rice also controls the number of leaves (Liu et al., 2009). Also, on the interval of Marker11517–Marker14862 of the QTL *qTN2-2* on chromosome 2 with a conditional QTL that controlled PH at several developmental stages in two environments, the high genetic variation of PH was explained (Che et al., 2020), as well as on the interval of Marker10138–Marker53481 of *qTN3-5*/*cQTN3-5* on chromosome 3 with a QTL affecting ear stem length (ESL) in different years and environments (Che et al., 2018). These regions may contain several QTLs controlling TN, PH, and ESL, respectively, or one QTL affecting these traits meantime. Therefore, further studies on these regions can provide a reliable basis for improving the breeding efficiency of *A.* Gaertn. The continuous QTL in this study that affected TN at different stages could also be regarded as the multiple effects of QTL by temporal. Such multifunctional QTL needs to be finely mapped in future studies to provide a basis for assistant breeding of valuable traits and joint breeding of multiple excellent traits of the *Agropyron* plants.

### The Temporal Expression Characteristics of Tiller Number

There was no QTL for TN that could be detected in every period, and some QTL was expressed in several periods (Figure 3), such as major QTLs *cQTN1-3, cQTN1-5, cQTN4-1, qTN1-4*, and *qTN5-1*, which were all detected more than once and controlled different stages of TN each time. This implies that the QTL expression selectively had different effects at different stages that showed the characteristics of time expression. In this study, the trend of TN development rate performed as increased rapidly at first stage then decreased slowly and increased gradually at last stage, which showed that the tiller of *A.* Gaertn. was more active at RS-JS than HS-MS. Meanwhile, the number of QTLs detected at RS-JS was greater than HS-MS. Thus, the number of QTLs may be related to the tiller rate. This may be because by the late stages of the *A*. Gaertn. growth, many nutrients were transported to the reproductive organs, caused the tiller bud no more born even the died of tillers, so the TN decreased gradually (Shang et al., 2021).

### Comparative Conditional and Unconditional Analysis Methods

Compared to the two methods of QTL analysis, conditional analysis detected more QTLs than unconditional analysis, and eleven and eight QTLs were detected by conditional or unconditional analysis methods, respectively; other nine QTLs were identified by two analysis methods. This may be the effect of these conditional QTLs being faint that not reaching significant levels and could not be identified by unconditional analysis. Conversely, some QTLs may have been expressed with small effects being undetectable but accumulated to a certain period, of which they are sufficient to be identified as unconditional QTLs. The combination of conditional and unconditional methods can detect more QTLs than the unconditional method only, which means that more alternative loci can be provided for marker-assisted breeding.

Notably, located in the same interval, *cQTN3-1* (TMTH) and *qTN3-1* (TM) controlled TN in 2017, but the PVE of *cQTN3-1* (13.85%) was greater than that of *qTN3-1* (9.39%). A similar situation was found for *qTN3-2* and *cQTN3-2* and also for *qTN3-5* and *cQTN3-5*. Generally, the PVE of a certain QTL indicated the ratio between the variance induced by the QTL and the total phenotypic variance. This contradictory result may be caused by the large differences in the total variance of TN at different stages. Another possibility is that there are some negatively expressed QTLs before the HS with very weak undetectable effects and offset part of the effect of *cQTN3-1*, resulting in a reduced cumulative effect, so that the PVE of *qTN3-1* is larger than that of *qTN3-1* (Tian et al., 2011). This implies that TN is a continuous process, and the effects of the same QTL will change with time.

### Relationship Between Quantitative Trait Locus of Tiller Number of *Agropyron* Gaertn. and the *Triticeae* Species

A new tillering regulation gene that inhibited the growth of tillering buds was fine mapping in 0.35 cM interval on chromosome 2DL of wheat (Wang et al., 2021). In this study, a stable major QTL *qTN2-3/cQTN2-4* was also detected on chromosome 2. In addition, since the *A*. Gaertn. is a homologous species of wheat, three QTLs were found at the collinearity intervals by comparing the maker sequences of seven linkage groups of *A*. Gaertn. with the genomic sequences of wheat. The unconditional QTL *qTN3-4* was detected at Marker53481 on chromosome 3, which corresponds to wheat 3DS_2575113. The unconditional QTL *qTN2-2* was detected at Marker11517 on chromosome 2, which corresponds to wheat 5DL_4543085. The conditional QTL *cQTN1-4* was located at Maker4585 on chromosome 1, corresponding to wheat 1DL_2269856 (Zhang et al., 2015).

Compared with the barley, two QTLs could have corresponded to the collinearity interval of barley; one conditional QTL *cQTN1-4* was located at Marker4585 on chromosome 1, which corresponding to barley morex_contig_1638559; and one unconditional QTL *qTN2-2* was detected at Marker11517 on chromosome 2, which corresponding to barley morex_contig_79233 (Zhang et al., 2015). Most of the loci had no corresponding relationship between wheat and barley, indicating that the genome of *A.* Gaertn. might be quite different from that of the *Triticeae* species, but these corresponding relationships may provide a basis for gene transfer in the future.

## Conclusion

In total, 37 QTLs for TN were detected by unconditional and conditional QTL mapping method in 4 years. A total of 12 major unconditional QTLs and 16 major conditional QTLs for TN were located. Most of the QTLs expressed at one developmental stage, unconditional major QTLs *qTN1-4* and *qTN5-1*, conditional major QTLs *cQTN1-3*, *cQTN1-5*, and *cQTN4-1* were detected more than once. Four relatively major stable conditional QTLs were detected in 2 years. In this study, conditional and unconditional QTL methods were combined to describe the development of tillering of *A.* Gaetrn. more comprehensively, and the temporal expression of these TN-related QTLs was revealed. This study brings an in-depth perception of the genetic basis of TN, as well as helpful to the utilization of forage resources.

## Data Availability Statement

The original contributions presented in the study are included in the article/supplementary material, further inquiries can be directed to the corresponding author/s.

## Author Contributions

YC, WL, and LL designed the research. YC, YH, NS, and YY performed the research. YC, YH, and NS wrote the manuscript. LW, XY, YZ, JZ, HH, XL, and SZ participated in the preparation of the reagents and materials in this study. All authors contributed to the article and approved the submitted version.

## Conflict of Interest

The authors declare that the research was conducted in the absence of any commercial or financial relationships that could be construed as a potential conflict of interest.

## Publisher’s Note

All claims expressed in this article are solely those of the authors and do not necessarily represent those of their affiliated organizations, or those of the publisher, the editors and the reviewers. Any product that may be evaluated in this article, or claim that may be made by its manufacturer, is not guaranteed or endorsed by the publisher.
